# Biomechanical modeling of cell chirality and symmetry breaking of biological systems^☆^

**DOI:** 10.1016/j.mbm.2024.100038

**Published:** 2024-01-05

**Authors:** Tasnif Rahman, Frank D. Peters, Leo Q. Wan

**Affiliations:** a Department of Biomedical Engineering, Rensselaer Polytechnic Institute, Troy, NY 12180, USA; b Center for Biotechnology & Interdisciplinary Studies, Rensselaer Polytechnic Institute, Troy, NY 12180, USA; c Department of Biological Sciences, Rensselaer Polytechnic Institute, Troy, NY 12180, USA; d Center for Modeling, Simulation, and Imaging in Medicine, Rensselaer Polytechnic Institute, Troy, NY 12180, USA

## Abstract

Accumulating evidence strongly suggests that cell chirality plays a pivotal role in driving left-right (LR) symmetry breaking, a widespread phenomenon in living organisms. Whole embryos and excised organs have historically been employed to investigate LR symmetry breaking and have yielded exciting findings. In recent years, *in vitro* engineered platforms have emerged as powerful tools to reveal cellular chiral biases and led to uncovering molecular and biophysical insights into chiral morphogenesis, including the significant role of the actin cytoskeleton. Establishing a link between observed *in vivo* tissue chiral morphogenesis and the determined chiral bias of cells *in vitro* has become increasingly important. In this regard, computational mathematical models hold immense value as they can explain and predict tissue morphogenic behavior based on the chiral biases of individual cells. Here, we present the formulations and discoveries achieved using various computational models spanning different biological scales, from the molecular and cellular levels to tissue and organ levels. Furthermore, we offer insights into future directions and the role of such models in advancing the study of asymmetric cellular mechanobiology.

## 1 Introduction

Chirality is a fundamental geometric concept that represents structural asymmetry. An object is considered chiral if the object and its mirror image are not identical, *i.e.*, the object and its mirror image cannot be superimposed. The most common example of chirality is our hands – in fact, the Greek word for “hand” serves as the etymological root of the word “chirality”. Biological systems are inherently chiral owing to the chiral organic molecules that serve as their building blocks, such as amino acids and sugars [1]. Common examples of chiral structures in nature include the twining of climbing trees, the helical nature of snail shells, and human internal organs such as the heart and stomach. Recent work has shed significant light on the biological and physical mechanisms that regulate large-scale chiral structures in biological systems [[2], [3], [4], [5], [6]]. Particularly, chirality has been studied extensively in members of the clade Bilateria, owing to their externally bilaterally symmetric bodies contrasted by several asymmetric organ systems – such as the heart and gut [[3], [4], [5],^7^]. At the molecular scale, the chirality and associated chiral centers of carbon-based organic molecules have long been known and very well studied. Brown and Wolpert hypothesized that the development of LR asymmetry of organism is derived from a hypothesized chiral F molecule which can be arranged along the antero-posterior (AP) and dorsoventral (DV) body axes to drive LR symmetry breaking [8]. However, the relationship between molecular chirality and chiral phenomena at large length scales is still poorly understood. The chirality at the intermediate cellular level, connecting molecular chirality and tissue chirality, has caught the attention of researchers in recent years.

Recent studies using *in vitro* models have revealed that cells are themselves chiral [3,4,[9], [10], [11]]. Work from several groups has shown that eukaryotic cells demonstrate chiral alignment with respect to tissue boundaries, with individual cells undergoing directionally biased migration at the boundaries *in vitro* [9,10,[12], [13], [14]]. Similar biased alignment and migration are also observed in micropattern-based vascular models [15]. Preferred handedness was also observed in individual cells spontaneously rotating in a 3D mechanical gradient, self-organizing their actin cytoskeleton in chiral swirls on isolated 2D protein islands, and even in the patterns of neuronal growth cone development [[16], [17], [18]]. Interestingly, the chiral alignment of micropatterned cells observed in *in vitro* tissues is reminiscent of the chiral alignment of cells observed in LR asymmetric tissues *in vivo* such as the heart myocardium before the initiation of c-looping, the alignment of epithelial cells in the embryonic gut tube also before looping, and the epithelial cells making up the chirally rotating drosophila genitalia [3,4,10,19]. A significant role for the actin-cytoskeleton and its associated regulatory framework in establishing cell chirality has been revealed in these efforts, lending credence to the idea that large-scale chiral phenomena can be derived from cell chirality. Indeed, cell chirality has emerged as a crucial regulator of LR asymmetric tissue and organ morphogenesis [4,5,11].

A biomechanical understanding of chiral tissue morphogenesis is required for the field to move forward. Therefore, much recent work has focused on creating mathematical models to describe the mechanical behaviors of chiral morphogenesis processes. While *in vitro* techniques such as traction-force microscopy and novel nanowire magnetoscope technologies have been used to measure chiral forces generated by cells, significant efforts are still necessary, if possible, for them to be used for studying the multicellular behavior of chiral morphogenesis [[20], [21], [22]]. As no efficient experimental techniques are currently available to determine the mechanical biases of individual cells within *in vivo* tissues, scientists have been comparing chiral tissue morphogenesis to non-living systems such as liquid crystal nematics and particulate systems. Understandably, the biomechanical models provide several features that *in vitro* or *in vivo* experimental approaches often cannot offer [16,19,21,23]. First, they allow for the prediction of tissue morphogenetic behavior based on specific cellular mechanical properties and tissue geometry. Second, through sensitivity analysis of model parameters, these models can identify major factors that regulate chiral morphogenesis. Third, these models can facilitate the identification of biophysical origins of cell chirality by comparison of the observed chiral phenomenon with the model prediction [23]. Finally, they can help define simple quantitative measures that can be used for the characterization of the observed seemly complicated morphogenetic behavior [9,23]. Thus, computational modeling has emerged as an obvious, yet exciting tool for studying cell chirality, which involves the chirality of mechanical forces at various biological scales.

In this paper, we will review recent progress in the mathematical and mechanical modeling of cell chirality at all biological scales. We will start with molecular modeling of cell chirality, focusing on the actin cytoskeleton. We will then present the modeling of cell chirality in multicellular systems and the potential biomechanical role of cell chirality in chiral tissue morphogenesis.

## 2 Modelling chirality at the molecular level

Most biological molecules are chiral in nature. For example, most amino acids exist as their L-enantiomer, whereas sugars are mostly found in their D-enantiomeric form – this phenomenon is called homochirality and is conserved among all life [1]. The proteins involved in determining the shape and migratory behavior of eukaryotic cells are also chiral in their structure and function. It is widely accepted that the actin cytoskeleton plays a pivotal role in LR symmetry breaking in cells [1,9,[15], [16], [17]]. For instance, the inhibition of actin polymerization can perturb the chiral behaviors observed in cells and tissues, such as the chiral alignment of cells on micropatterns [9]. Similarly, myosin activity has also been identified as a driving force of chiral morphogenesis, likely due to the chiral nature of myosin motor activity – such as that of Myosin 1D found in Drosophila [24,25]. Furthermore, the actin cytoskeleton itself demonstrates chiral behavior in its dynamic self-organization in cells subject to isotropic confinement [16,20]. Chiral actin dynamics have also been shown to drive chiral alignment of microtubule structures, biased positioning of the microtubule organizing center, and biased motion of the cell nucleus [4,16,26,27]. Therefore, in this section, we will discuss the mathematical models developed to describe the cytoskeletal mechanics behind this phenomenon, focusing on the progress made in elucidating the role and mechanism of actin-related symmetry breaking within cells.

### 2.1 Chiral cortical actin flow

**Fig. 1:**
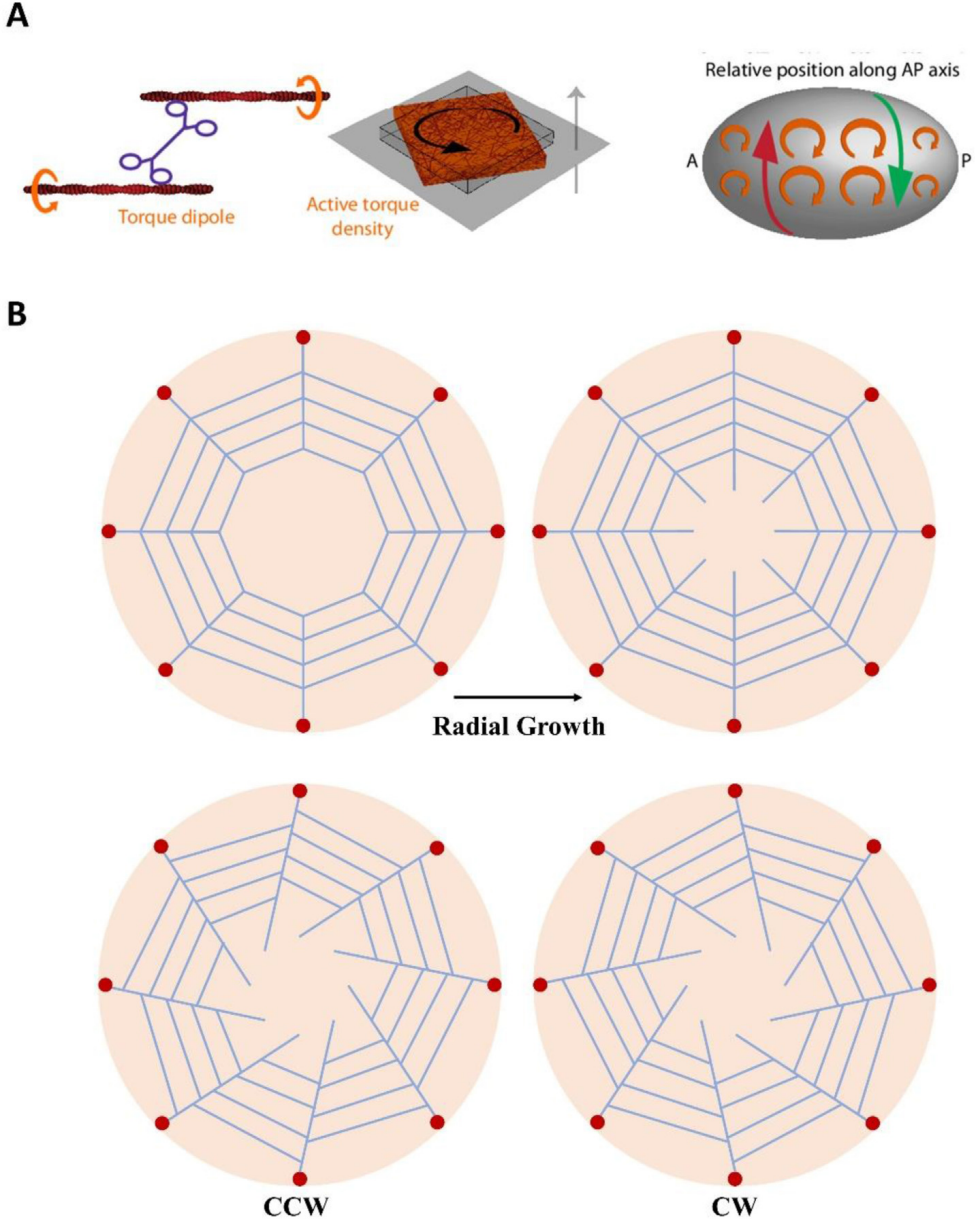
**Physical Models of Cell Chirality at the Molecular Level. A.** Schematic showing myosin driven torque dipole generation within actin fibers (left) and the bidirectional actin flow along the AP axis of the *C. elegans* embryo (right). Adapted from Naganathan et al., 2012 under the Creative Commons Attribution License (CC BY) [11]. **B.** Schematics of chiral radial actin fiber swirling.

Fürthauer et al. modeled cortical actin as thin films of active chiral fluids – with chiral stresses being generated by nematic simple motors. These motors are derived from elementary torque dipoles, which mimic those generated by myosin motors bound to two antiparallel actin fibers. Using such a hydrodynamic approach to defining actin flows, the authors demonstrate large-scale fluid flow, and chiral dynamics derived from molecular torques [28,29]. This suggests that such asymmetries in flow can lead to cellular level-chirality – as observed within developing the dividing *Caenorhabditis elegans* embryos [30]. Naganathan et al., first described the chiral dynamics of the actomyosin cytoskeletal machinery in the *C. elegans* embryo with the help of a thin chiral fluid flow model of the actin cytoskeleton [11]. The authors observe a biased flow of fluorescently labeled actin structures across the AP axis of the embryo and develop a physical model to describe observed chiral flow behavior – by modeling the actomyosin cortex as an active, thin chiral fluid (Fig. 1A) [11]. They define the AP ($v_{x})$ and the orthogonal ($v_{y}$) flow velocity components as being respectively driven by active tension and active chiral torque generated by ATP driven actomyosin activity as described below.

(1) $$\partial T/\partial x=\eta \partial^{2}v_{x}/\partial x^{2}-\gamma v_{x}$$

(2) $$\partial \tau /\partial x=\frac{1}{2}\eta \partial^{2}v_{y}/\partial x^{2}-\gamma v_{y}$$

Here, the tension T along the AP axis and the torque density τ define a chiral index c=τ/T, η is the two-dimensional viscosity of the cortex, and γ refers to the friction in the cell membrane and cytosol [11]. The authors assume that torque and active tension are both linearly proportional to local concentrations of myosin. The tension force is derived from the pull generated by myosin heads connecting neighboring actin filaments, while the torque force is generated by the twisting of the actin filaments during the ATP-driven myosin head contraction. At scale, these molecular forces combine to cause a contraction and rotation of the cortex. To verify the accuracy of their model, they quantified the flow of the actomyosin cortex using particle image velocimetry and showed that while most flow occurs in the AP axis, there is a small orthogonal component of the flow field as well with a counter-rotating behavior between the anterior and posterior axes (Fig. 1A). Furthermore, the authors noted that this molecular symmetry breaking precedes the observed rotation of these cells – suggesting that the chiral molecular flows drive chiral cellular rotations. The authors went on to show the myosin motor and RhoA dependence of this process. Myosin perturbation causes a decrease in the overall flow rate, but no changes in the chiral index are observed, while Rho signaling regulates only the chiral torque component thereby changing the value of c [11]. The authors then investigate the role of these chiral flows in multicellular organization and embryonic symmetry breaking. They observe the chiral alignment of cells in the 4-cell stage embryo as well. This bias is similarly regulated by Rho activity. Finally, perturbation of the overall body-axis determining Wnt signaling pathway revealed a reduction of chiral index within individual cells, suggesting that global LR signaling may regulate chirality at the cytoskeletal level. Similar concepts have been used to describe the ATP-driven motion of *in vitro* actin clusters [31].

Using a very similar model, Pimpale et al., discovered a lineage-specificity of the chiral flows within *C. elegans* embryos [32]. They tracked actomyosin flow in all cells during the first nine cell divisions post-fertilization. The authors show that chiral flows appear only on the anterior founder cells during cytokinesis, but no chiral counter-rotation is observed in the posterior stem cells or the endomesodermal precursor founder cell lineages at the same timeframe. Furthermore, with RNAi and fluorescence imaging experiments, the authors show that not only are the chiral flows quantified based on the chiral thin-film theory lineage-specific but they are also associated with skews in spindle formation and cell division orientation [32]. In fact, the counter-rotation velocity deterministically regulates the cell alignment skew – in accordance with a directional friction-based model where the rate of angular skew is proportional to the counter-rotation velocity, while the directionality depends on the relative value of the friction on either side of the inner eggshell walls. Interestingly, our group characterized distinct chiral biases cells from the three different germ lineages derived from human embryonic stem cells (hESCs), suggesting a strong phenotype dependence of cell chirality [6].

### 2.2 Chiral swirling of actin fibers

Tee et al. described single-cell chiral cytoskeletal dynamics *in vitro* using human foreskin fibroblasts micropatterned on circular fibronectin islands [16]. Under isotropic confinement within the micropatterns, the cells developed robust focal adhesions (FAs) with the fibronectin substrate and formed a distinct, and initially isotropic network of radial and transverse F-actin fibers (RFs and TFs). Over time, these networks were observed to undergo asymmetric self-organization which lead to the chiral counterclockwise (CCW) “swirling” of the RFs, in tandem with a centripetal flow of the TFs [16]. They also show that the actin crosslinking protein α-actinin-1 regulates the directionality of the twirling, with a significant increase in the number of clockwise (CW) swirling cells upon overexpression of the crosslinker. The authors also ruled out any role played by the microtubule network, in accordance with previous evidence [9]. How actin crosslinking and polymerization can lead to chiral swirling of the RFs, however, is not obvious. The authors thus develop a physical model to describe the actin fiber dynamics [16].

The model consists of a symmetric arrangement of radially aligned RFs as flexible rods, and TFs bound to adjacent RFs as contractile elastic rods (Fig. 1B). The contractile stress within the TFs is held constant for all TFs throughout the simulations. The RFs elongate radially by Formin-dependent actin polymerization, while new TFs are probabilistically generated during the simulations at the Formin end of the RFs. The centripetal component of the TF contractility moves the TF-RF contacts along the RFs, while the tangential component (in the circumferential direction) can cause RF bending (Fig. 1B). In the initial isotropic arrangement, the tangential forces are balanced. However, slight deviation of RFs from the radial alignment causes rearrangements of its associated TFs, leading to a progressively compounding symmetry-breaking event.

The authors further model the chiral rotation of growing RFs. In the wild-type regime, Formin is capped and immobilized - preventing rotation of the RF at the FAs but allowing for its rotation at the free cytosolic end. A+14° rotation occurs with each actin dimer addition and promotes a CCW RF alignment. If actin crosslinkers are present, the rotation at the cytosolic end is prevented, resulting in the accumulation of torsional stress which is relieved every 12th polymerization event by a −166° rotation, causing a CW RF alignment. The rotation of RFs causes friction generation at the RF-TF binding locations similar to a rack and pinion system, causing the TFs to slide tangentially, leading to a chiral RF swirling [16]. These two physical models of actin swirling lay the foundation for actin-driven torque generation within eukaryotic cells and hold significant implications for the chiral morphogenesis of tissues and organs.

Li and Chen subsequently model the rotation of RFs because of actin polymerization, and the role of α-actinin cross-linkers in switching swirling direction. In this molecular clutch model, single actin filaments are represented by rods which grow and rotate, along with the formin interface which is modeled as a torsional spring. This rotation is transferred to the RFs through α-actinin cross linkers which are also modeled as torsional springs. When the filament forms cross-links with the RF, both rotate in a positive direction. Subsequently, the filament is capped, detached from Formin, and incorporated completely into the RF. This causes the stored elastic energy to be released and causes the RF and filament to rotate negatively. The difference between the two angles of rotation determines the overall rotation and swirling of the RF.

The authors show that high crosslinker attachment rate, high crosslinker concentration, and high actin monomer concentrations all lead to an overall CW swirling - consistent with *in vitro* findings and previous computational predictions [27]. Li and Chen further developed this model by introducing a sliding mode of crosslinker mobility along the growing filament, i.e., the crosslinker is allowed to move along the filament [33]. They compare this model to one where the crosslinkers remain fixed to show that the sliding motility mode leads to CW, CCW and no swirling cells whereas the fixed mobility mode does not lead to CW swirling – suggesting a sliding motility may be more realistic [33]. Finally, the authors extend the model to investigate three different modes of torque relaxation at the Formin-end of filaments. However, none of the modes yield satisfactory predictions based on experimental data [34]. Further studies may reveal more specific molecular mechanics of chiral RF swirling.

These models provide a more detailed physical understanding of the role of actin polymerization in generating intracellular torque and describe the specific role of actin crosslinkers and their mechanisms of action in determining the directionality of actin swirling. However, they still have limitations. Firstly, the rotation of RFs is not used to model the swirling of the overall actin structure itself in the Tee et al. model. Secondly, both the Tee et al. and Li and Chen models consider actin fibers as simple rods, which allows for a straightforward model for actin-generated cellular torque. However, more detailed models of actin dynamics are available in the field. One such example is the Cosserat Theory-based model of actin polymers reported by Floyd et al. [35]. The group further enhance their model by including an ellipsoid model of actin polymers which considers actin monomers (the ellipsoids) and their specific chemical bonds with neighboring monomers in the follow-up study by Gunaratne et al. [36]. Incorporating such detailed actin dynamics into the models of swirling would more accurately represent the rotation of actin RFs and their subsequent contribution to overall swirling dynamics – with results comparable to mechanical measurements performed *in vitro* [20].

## 3 Modelling chirality at the multicellular level

We have discussed how asymmetries in molecular actin dynamics can lead to cellular chirality and generate asymmetric torque forces. In the next sections we discuss efforts in elucidating how cellular and molecular level asymmetric forces can lead to symmetry-breaking at the multicellular scale. This is of particular interest since aberrant development and maintenance of tissue asymmetry can lead to disease [[37], [38], [39]]. We describe several different methods of simulating the establishment of asymmetric tissue and organ morphologies using vastly different mathematical approaches.

### 3.1 Reaction-diffusion model

**Fig. 2:**
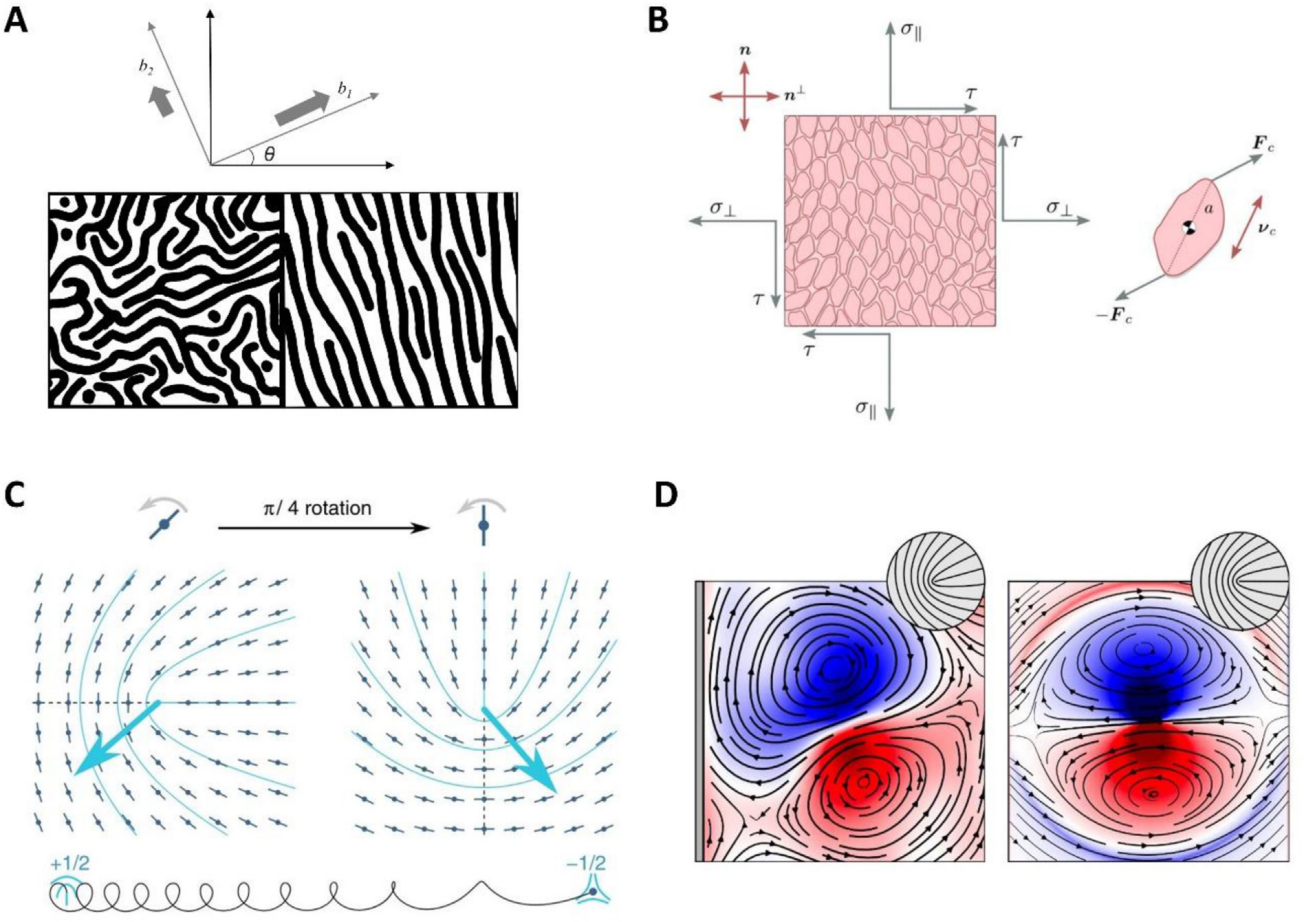
**Diffusion and Nematic Models of Multicellular Chiral Morphogenesis. A.** Schematic showing the factors b1 and b2 that determine migration rates of cells along the given orthogonal directions depicting an asymmetric setup where b1>>b2 (Top). Simulation results depicting model tissue aggregates under isotropic conditions where b1 ​= ​b2 (bottom left), and asymmetric conditions (bottom right). Schematic based on Chen et al., 2011 [11] **B.** Stress contribution in a volume element parallel and perpendicular to the director **n** of a nematic model is shown (left). The chiral stress **τ** generated by a single cell is assumed to arise as a force dipole acting on opposing ends of a given cell (right). Adapted from Hoffman et al., 2019 under the Creative Commons Attribution License (CC BY-NC 3.0) [41]. **C.** Chiral nematic model showing rotation of a +1/2 topological defect due to rotations of individual cells (top). +1/2 defects are also seen to preferentially migrate towards −1/2 defects during migration (bottom). Adapted from Maitra and Lenz, 2019 under the Creative Commons Attribution License (CC BY 4.0) [44]. **D.** Schematic showing the cellular flow surrounding +1/2 defects close to (left) and far away from (right) a boundary (depicted as gray bar on left side of image). Adapted from Yashunsky et al., 2022 under the Creative Commons Attribution License (CC BY 4.0) [45].

Biological tissues often demonstrate behavior analogous to inanimate physical systems. A common example of this is that biological systems often involve signaling molecules that follow reaction-diffusion dynamics. Chen et al., describe a reaction-diffusion model that predicts the formation of chiral, parallel cell aggregation patterns following growth in alternating stripe-shaped micropatterns [15]. Upon removal of intermediate non-adherent stripes, the cells demonstrate chiral migration and wound-healing behavior. Upon long-term culture following chiral alignment, coherent and parallel cell aggregation is observed, compared to labyrinthian patterns observed in non-patterned control cultures [15]. This behavior is accurately recapitulated in a combined reaction-diffusion and chemotaxis model driven by the migration activator BMP-2 and its faster diffusing inhibitor MGP – with the assumption being that cells preferentially migrate towards areas of the higher activator morphogen. The authors use previously described reaction diffusion dynamics to determine morphogen patterns and combine it with a chemotaxis model which introduces two tunable vector variables that describe the differential migration in the two principal axes [15]. When anisotropies are introduced by setting the migration along one axis several orders of magnitude larger than the orthogonal axis, and biased by an angle θ, the resulting cellular aggregates form parallel aggregates as observed in the micropatterned *in vitro* samples (Fig. 2A). In contrast, an isotropic model with equal migration on both axes leads to a labyrinthian pattern of cell aggregation compared to the biased model (Fig. 2A) The authors further show that the observed chiral behavior is dependent on the accumulation of stress fibers at the interface boundaries – and that prevention of these interactions attenuates LR polarity [15]. They show that the microtubule organizing center polarizes at the boundary edge, like the leading-edge cells of wound healing assays, and that the cell-substrate interactions are required for this polarity and the subsequent chiral cell cluster formation [15]. Similar results were obtained by Li et al., who showed chiral symmetry breaking in a reaction-diffusion system under the influence of an external chiral electric field [40]. While different from the chemotaxis reaction-diffusion model, this study also demonstrates that an active external asymmetry can induce symmetry breaking within otherwise symmetric reaction-diffusion systems.

### 3.2 Continuum mechanics models

To study the collective behavior of cells, biophysicists often draw inspiration from non-living collective systems [[41], [42], [43]]. These non-living systems and their associated theories have proven quite useful for predicting collective tissue behaviors. Active matter systems of liquid crystals display similarities to developing cellular monolayers. In liquid crystals, rod-like particles are arranged in a fashion such that their orientational order is preserved. Further studies of chiral active matter revealed differing forms of asymmetry arising from antisymmetric stresses [28,44].

Duclos et al. study the spontaneous flow of cells in confined patterns as they relate to stresses generated by liquid crystals [45]. They highlight that cells follow a conserved alignment direction within the pattern boundaries and theorize that the degree of tilting is controlled by the strength of chiral stresses induced by biased cellular motion. A continuum model is utilized for the purposes of predicting this behavior. These models assume that cellular motion is very slow and viscous, generating an overall incompressible flow regime (*i.e.*, ∇·v=0). For an incompressible fluid flow, the Navier–Stokes equation for cellular motion can be simplified so that the stresses within the monolayer can be better extrapolated. Studies attempting to model cellular motion as a flow of aligned particles require a robust definition of the active stresses being generated and the Frank free energy of the system. The Frank free energy describes the alignment and polarization interactions between elongated particles within the system [46]. Hoffman et al. build upon the work completed by Duclos et al. to generate a thorough description of the active stresses experienced by cells within a monolayer below [47]:

(3) $$\sigma^{a}=\sigma_{∥}nn+\sigma_{?}n^{?}n^{?}+\tau \left(nn^{?}+n^{?}n\right)$$

Here, *σ*_∥_ and *σ*_?_ represent the stresses experienced by some volume element in the parallel and perpendicular directions in respect to nematic director **n** respectively. The new value **τ** describes the contribution of the chiral cellular rotation to the active stress arising from an intrinsic force dipole (Fig. 2B). With this chiral component to the active stress added, examination of the alignment angle within stripe patterns with increasing widths sees improved comparison with experimental data [45].

Another hallmark of liquid crystals are topological defects in which the perfect alignment of particles within a layer is disrupted. Cellular monolayers of Neural Progenitor cells (NPC) and Madin–Darby Canine Kidney (MDCK) cells, for example, have displayed such topological defects [48,49]. Hoffman et al. continue with their description of chiral active stress to describe the motion of +1/2 (“comet like”) disclinations. In monolayers of extensile or contractile particles, +1/2 defects will move in the direction of their head or tail respectively. In the presence of a chiral particle, as cells have been observed to behave as, it is predicted that the defect will rotate along its path of motion. Hoffman et al. provide an equation below to relate angle of biased migration to the active stress and the chiral torque:

(4) $$\Theta_{tilt}=\arctan\left(\frac{2\tau }{\sigma_{∥}-\sigma_{?}}\right)$$

The angle of defect rotation is related to the overall chiral torque if the overall active forces are known. This framework provides many similarities to one study completed by Maitra and Lenz [50]. Maitra and Lenz simulate positive half-integer defects of a nematic material to migrate and rotate in respect to negative half-integer defects within the system, leading to a recombination event (Fig. 2C). Yashunksy et al., utilizing a similar model to Hoffman et al., demonstrate that +1/2 topological defects in close proximity to boundaries will preferentially rotate while defects far away do not (Fig. 2D) [51]. Chiral flow on edges of patterned surfaces has been observed on stripe shaped domain for several different cell types (i.e. MDCK and NPC) [10,52]. Yamauchi et al. specifically apply stochastic modeling of active nematic particles on boundaries. They determined that steady state flows induced by geometry are hallmarks of chiral active nematics.

In contrast to half-integer defects, chiral events of integer defects have also been studied extensively in the presence of boundary conditions. Blanch-Mercader et al. examine the mechanics and flows of integer defects created by C2C12 cells grown on circular micropatterns [53]. Here, cells align in spirals on these micropatterns in directions consistent with previous alignment studies of C2C12s [9]. Blanch-Mercader et al. additionally apply continuum dynamics assuming incompressible flow of cells. While their model does not assume the presence of an active chiral stress, initial conditions of the model assume that cells are already aligned in a biased fashion. In another study by Hoffman et al., the model is expanded to examine the 3D morphogenesis of spiral aligned cells [54]. Here, researchers were able to recapitulate the morphogenesis observed *in vitro* by Guillamat et al. [55].

While nematic modeling of cellular motion has proved effective for predicting cellular behavior, there are situations when it fails. Since cells are only modeled as rods, the amorphous structure of the boundaries and cytoskeleton are lost. This leaves these models better suited for studying very elongated cells or very large populations of cells, while mechanical properties of individual cells are largely ignored.

### 3.3 Vertex models of chirality

Like nematic models, vertex models commonly used to simulate the dynamics of non-living systems like foam have gained popularity with its use in multicellular tissue dynamics [43,[56], [57], [58]]. In this model, instead of nematic rods, cells are modeled as polygons defined by their vertices and connected by edges which represent the cell–cell junctions. The dynamics of the system are then defined by defining energies based on polygon geometry and applying forces on the polygon vertices. These forces are then used to displace the vertices – usually using a gradient descent energy minimization algorithm or a simple Euler method, depending on the nature of the simulations. Generally speaking, these models incorporate polygon area and perimeter elasticities, to represent the forces generated by the actomyosin cortex and cell–cell adhesions. Some models also incorporate a line-tension which represents the energy associated with the cell membranes. It is possible, however, to include further intricacies by adding novel energies, forces, or dynamic regulations of their associated parameters. Details of various such methods are reviewed elsewhere [43,56]. In what follows, we discuss several implementations of chiral biases using some variations of the vertex model described above.

#### 3.3.1 LR asymmetric line-tension

**Fig. 3:**
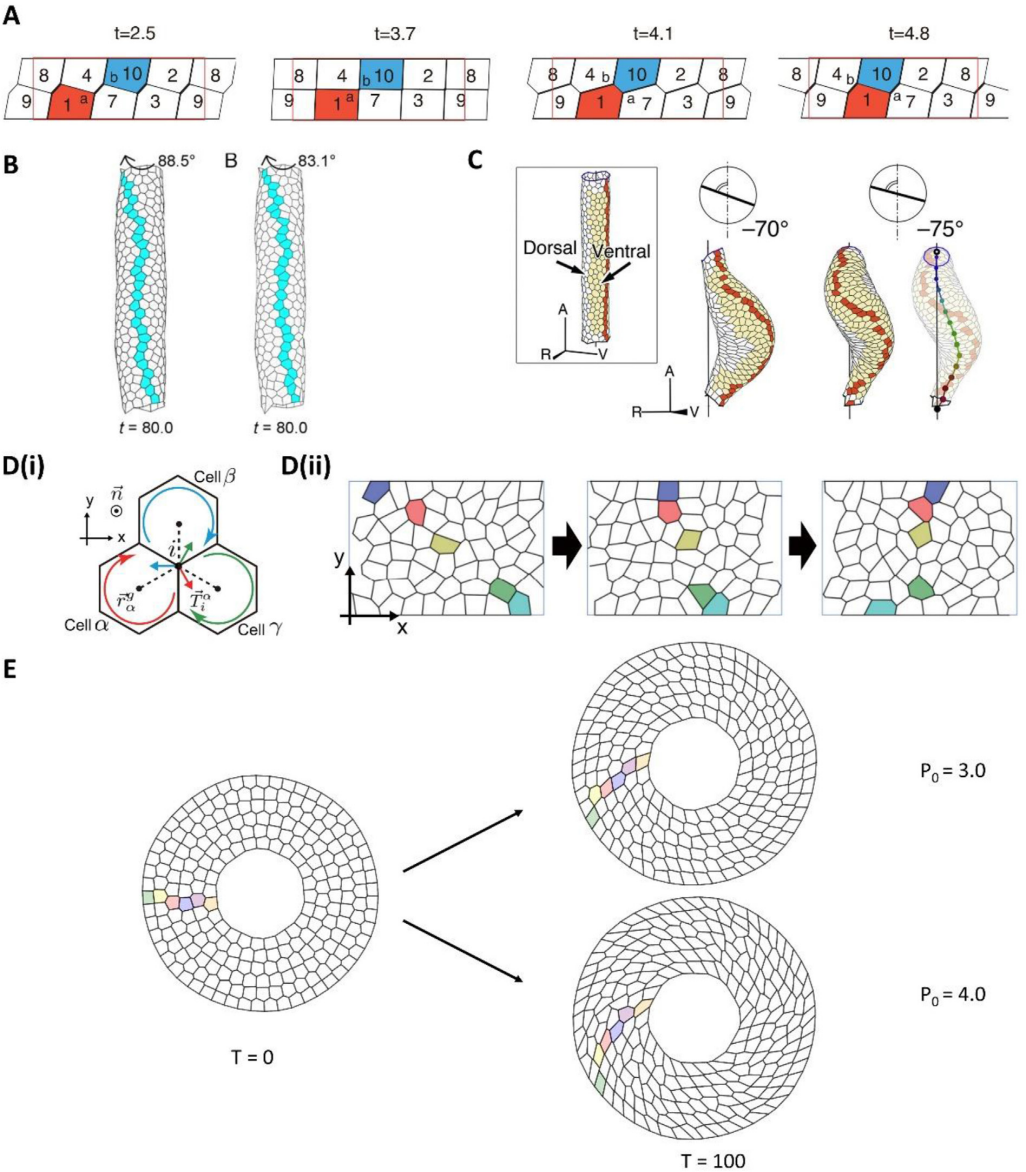
**Vertex models of chiral morphogenesis. A.** Bidirectional migration of vertex model cells at opposing boundaries, showing cell-neighbor exchange leading to tissue sliding. Adapted with permission from Sato et al., 2015 [60]. **B.** 3D Vertex model of looping hind-gut showing rotation of the tissue. Adapted from Inaki et al., 2018 under the Creative Commons Attribution License (CC BY 4.0) [5]. **C.** 3D Vertex model of looping heart-tube, showing the looping dynamics under different biased line-tension directions. Adapted from Honda et al., 2021 under the Creative Commons Attribution License (CC BY 4.0) [57]. **D. (i)** Schematic depicting the application of chiral torque on a 3-cell vertex model unit and **(ii)** Bidirectional migration of cells at the boundaries of a periodic tissue with two fixed boundaries with cells subject to a clockwise torque force. Adapted from Yamamoto et al., 2020 under the Creative Commons Attribution License (CC BY 4.0) [20]. **E.** Time-series images of ring-shaped vertex model tissues, showing different migration under different jamming states: solid (top, p_0_ ​= ​3.0); and fluid-like (bottom, p_0_ ​= ​4.0); unpublished figures [59].

Experimental data from *in vivo* chiral tissues have shown that when cells within tissues take on a biased alignment relative to the longer axes of tissues, the junctions which align parallel to the biased cellular alignment direction demonstrate stronger junctional protein and actomyosin expression. This increased expression is associated with a higher tension value [3,4]. Such biases have been observed in various organ systems, including *Drosophila* hindgut and genitalia, as well as the looping chick embryonic heart [4,5,19]. Similarly, polarized junction localization is also observed in non-LR asymmetric, but anisotropic convergent extension events during tissue elongation [59]. Thus, it stands to reason that biased tension could lead to chiral cell alignment. To test this idea, Sato et al. employ a vertex model strategy [60]. They model a strip like tissue with physical boundaries in the y-direction, representing the tissue boundaries, and Lees-Edwards periodic boundary conditions in the x-direction (Fig. 3A). They define the cell dynamics as described above, however, the average line-tension energy density value is set to be a function of the alignment angle of a given cell–cell junction, as defined below [60]:

(5) $$\gamma_{ij}\left(t\right)=\hat{\gamma }\left(\gamma_{0}+\gamma_{1}\;\cos\;2\left(\theta_{ij}\left(t\right)-\;\theta_{0}\right)\right)+\xi_{ij}\left(t\right)$$

Here, $\gamma_{ij}$ is the line-tension energy density, $\gamma_{0}$ and $\gamma_{1}$ are positive constants, $\theta_{0}$ is the angle between an edge and the physical boundaries at which the tension takes a maximum value and $\theta_{ij}$ is the angle associated with the specific edge connecting vertices *i* and *j*, and $\xi_{ij}\left(t\right)$ is a colored, time-correlated Gaussian noise which adds stochasticity.

In such a model, junctions aligned in a particular direction relative to the tissue boundary have a higher junctional strength, which subsequently leads to cellular alignment (Fig. 3A) [60]. The authors show that a uniformly distributed biased tension leads to the asymmetric alignment of cells as observed *in vitro* and the nematic model described above. Additionally, the model recapitulates the shear-like migration of the cells at opposing boundaries also observed in strip-like patterns *in vitro* (Fig. 3A) [60]. Spatial variations of this biased angle can lead to varied migratory properties, such as unidirectional migration. This data suggests that biases in line tension can drive collective cell migration. The group then extends these simulations and compares them to experimental data obtained from images of the *Drosophila* genitalia which undergoes chiral morphogenesis. They show that not only are the asymmetries dependent on actomyosin polarization *in vivo*, but that the biased line-tension can recapitulate the rotatory behavior of the developing genitalia within a ring-shaped vertex-model tissue [19]. Likewise, Hiraiwa et al., demonstrate similar asymmetric tissue flow and cell alignment in a ring-shaped tissue model – consistent with the asymmetric cell alignment and biased collective cell migration observed at the boundaries of *in vitro* micropatterned tissues [9,61]. The authors consider a polarization direction relative to the tissue boundaries and set the junctional tension higher for the edges most parallel to the polarization within each cell [61].

Taniguchi et al., performed similar experiments in the *Drosophila* hindgut, where the *Drosophila* E-cadherin (*D*E-Cad) is polarized in a similar direction dependent manner, relative to the AP axis [3]. They model asymmetric contractions like what was described above. However, they model the hindgut as a pseudo 3D tissue, which tracks 2D migration of a linear periodic tissue and artificially transposes them onto 3D cylinder. Even without any 3D migration, both the chiral alignment of the junctions and rotation of the tubular hindgut tissue were recapitulated [3]. To iterate and improve on this model, Inaki et al. create a similar model, but with a 2D tubular tissue in 3D space which allows for the 3D motion of each vertex (Fig. 3B) [5]. Translation to a 3D model requires several additional energy formulations to help maintain the 3D shape of the tissue were implemented. Specifically, energy terms are introduced to maintain the volume of the tissue, to maintain the circular boundary at either end of the tube, and to maintain the smooth surface of the tissue. Chirality is simulated by applying a higher weight to two opposing edges most parallel to a defined angle of polarity relative to the AP axis. The authors again recapitulate the twisting morphogenesis of the hindgut (Fig. 3B). However, the model still has limitations as it does not consider the growth of the tube during the symmetry-breaking process [5].

Finally, we briefly discuss a series of models used to determine the chirality of the heart tube during chiral looping reported by Honda [62,63]. The first model neglects cell-generated chiral forces but identifies the need for 3 axial asymmetries required to form a looped morphology [62]. The initial tube-like tissue is modeled as in Inaki et al., except they include cell divisions on the ventral side to reflect the growth of the heart tube. The asymmetries in the AP and DV axes are modeled using asymmetric growth on the ventral side of the tube, and an asymmetric lateral shift of the tube along the AP axis. However, for looping to occur, a third LR asymmetry is required. The authors model this as a biased torsional motion of the tube – which recapitulates *in vivo* morphogenesis. The torsion is applied to the fixed ends of the tubular tissue and thus serves as a bulk-chiral force [62]. In the following publication, Honda uses a similar chiral line-tension used previously by Inaki et al. instead of the bulk torsion and demonstrates that asymmetric line-tension can also lead to chiral looping (Fig. 3C) [63]. The model reveals that chirality is only required on the ventral side of the heart tube, as observed from cardiac myocytes from the looping chick heart tube, and that the ratio of left and right-biased cells determines the looping direction [4]. The model further demonstrates that directionally biased cell intercalations drive looping morphogenesis. Overall, these data suggest that asymmetries in the junctional contractile forces play an important role in chiral tissue morphogenesis.

#### 3.3.2 Chiral torque

Recent evidence suggests that chiral cells demonstrate biased rotation of the actomyosin cytoskeleton, as well as the cell body – potentially generating a chiral rotational force [4,16,17,20,64]. Thus, many in the field posit that the chiral rotation of individual cells is what drives chiral morphogenesis and vertex models to assess whether chiral torque can lead to chiral cell alignment have been developed. Yamamoto et al. define chiral torque as follows:

(6) $$\vec{T_{i}}=\sum_{\begin{array}{c} cell\;\alpha \;\\ about\;vertex\;i \end{array}}v_{\alpha }\left({\vec{r}}_{i}-{\vec{r}}_{g}^{\alpha }\right)\times \vec{n}$$

Here, $\vec{T_{i}}$ is the torque vector, *η* is a coefficient of friction, ${\vec{r}}_{i}$ is the position vector of the ith vertex, $\vec{n}$ is the normal to the tissue surface at vertex i, ${\vec{r}}_{g}^{\alpha }$ is the position vector of the area centroid of the cell α, v_α_ is a torque coefficient for cell α, and t is the time unit [23]. They show using a strip-like model tissue that chiral torque itself is sufficient to drive LR asymmetric migration and alignment (Fig. 3D(i,ii)) [23]. The rotation is modeled as a torque that causes either CW or CWW rotation of all the vertices associated with a cell. They also find a relationship between the migration velocity and the cell shape index, which determines the fluidity of the tissue – suggesting a potential role of the jamming state of cells on chiral behavior.

Using this model formulation and implementation of chiral torque, we have recently shown that chiral alignment on ring shaped micropatterns can be driven by chiral rotations of cells (Fig. 3E) [65]. We further show that the jamming state of the tissue, determined by the cell shape index P_0_ which is the ratio of the preferred perimeter divided by square root of the preferred area, regulates the chiral alignment. The jamming state also regulates the elongation and migration of the cells, with more fluid-like tissues undergoing more significant cell elongation, alignment, and bidirectional cell migration at the boundaries throughout the duration of the simulations (Fig. 3E) [65]. Further analysis of chiral properties and cell rearrangements will shed more light on the role of chiral torque in multicellular symmetry breaking. Recent work suggests that the enantiomorphic makeup of the cell population may lead to the looping of a tubular tissue [63], if enough of the population is biased in a particular direction – future work could elucidate how the distribution of chiral forces within cell populations, and complex tissue architecture effect chiral morphogenesis.

## 4 Concluding remarks

Recent progress in computational power and the development of computational tools for studying mechanical phenomena has driven a massive revolution in the way we study the mechanics of biological systems [41]. It is no surprise that many of these methods are now used to study cell chirality as well. While specific mechanisms of cell chirality development and regulation are still not fully understood, mathematical models are allowing researchers to investigate cell chirality and its mechanical and regulatory responses to various physical, chemical, and biological perturbations.

One common feature of most of these chirality models is that they draw inspiration from non-biological physical systems, whether it be rigid body mechanics, liquid crystal hydrodynamics, or the mechanics of particulate systems – this allows for researchers to characterize the chiral morphogenesis with just a few parameters. As a result, the model separates biomechanics from the complexity of the biological systems, and the model parameters will reflect the biophysical factors that regulate chiral morphogenesis. For example, in the vertex model, cells behave as elastic polygons, whereas in the nematic models, cell alignment forms topological defects, while the models developed for actin dynamics only consider the mechanical behavior of the actin fibers and associated crosslinkers and physical regulators. Interactions such as reaction kinetics, diffusion kinetics, stochasticity, and geometric confinement are needed for the hypotheses being tested [16,58,60,62,63,66]. Most of these models similarly allow for the simple and iterative modeling of chiral forces as well – a most exciting tool for studying cell chirality.

The models discussed currently have several important limitations, however. The inherent simplicity of these models is crucial in maintaining feasible computational speeds, which means that the introduction of further biological complexity may incur heavy processing costs. Similarly, multiscale modeling which considers all biological scales of morphology and regulation are not yet widely possible due to the immense computational power required. As a result, current models often only focus on individual mechanical and biological properties [43,56]. Future work should focus on improving the efficiency of modeling algorithms to allow for better real-time modeling and analysis, and the incorporation of multiscale modeling parameters. Additionally, advancements in *in vitro* and *in vivo* methods for measuring mechanical properties and dynamic forces, such as nanowire magnetoscopes, traction force microscopy and particle tracking that make these methods more robust and feasible is required to accurately verify future models, which will only increase in complexity as well [[20], [21]]. Thus, computational efficiency and more accurate real-world verification tools represent the biggest challenges facing the mathematical models of chirality. Finally, the biological regulation of chirality is not very well understood, thus making it difficult to develop substantial hypotheses to test using these models – so a more holistic understanding of the molecular and cellular bases for chiral force generation will also greatly improve the usefulness of these models. Ultimately, the goal of the field, in our view, should be the development of models that simulate the mechanics of chiral organ and tissue morphogenesis at every biological scale simultaneously, such that multiscale effects of regulation at each level may be studied, i.e. combining the mechanics at the molecular, cellular and tissue/organ levels with biochemical regulation and external mechanical perturbations among other relevant factors such as genetics. We believe such models would be more clinically relevant and allow us to accurately study cell chirality in the context of development and disease [37].

## Ethical approval

This study does not contain any studies with human or animal subjects performed by any of the authors.

## Declaration of competing interest

The authors declare that they have no known competing financial interests or personal relationships that could have appeared to influence the work reported in this paper.
